# Calcium vitamin D_3_ supplementation in clinical practice: side effect and satisfaction

**DOI:** 10.1186/s40200-016-0227-9

**Published:** 2016-03-10

**Authors:** Maryam Sanaei, Mohammad Banasiri, Gita Shafiee, Mahsa Rostami, Saba Alizad, Mehdi Ebrahimi, Bagher Larijani, Ramin Heshmat

**Affiliations:** 1 Chronic Diseases Research Center, Endocrinology and Metabolism Population Sciences Institute, Tehran University of Medical Sciences, Address: # 111, 19th St, North Kargar Ave, Tehran, Iran; 2 Orthopaedic surgery department, Medical school, Golestan University of Medical Sciences, Tehran, Iran; 3 Endocrinology and Metabolism Research Center, Endocrinology and Metabolism Research Institute, Tehran University of Medical Sciences, Tehran, Iran

**Keywords:** Calcium, Vitamin D, Side effect

## Abstract

**Background:**

The objective of this study was to assess side effects and satisfaction about OsteoCalVitFort (500 mg calcium and 400 I.U. of vitamin D3) usage.

**Methods:**

A total 186 people were participated with range age from 18 to 65 years old. Each participant received 1 pack that contains 60 OsteoCalVitFort tablet and used two tablet OsteoCalVitFort daily (1 tablet after breakfast and 1 after dinner). By a phone call, side effects and satisfaction about OsteoCalVitFort were assessed.

**Results:**

The rate of constipating (8.0 %) and bloating (12.5 %) were decreased significantly after OsteoCalVitFort supplement intake (1.2 %, and 0.6 %, respectively). Similar results were observed in metallic taste in mouth, tiredness, weakness, loss of appetite, bone/muscle pain and mental/mood change after Calcium Vitamin D3 supplementation intake. Totally, 94 % of patients were satisfied about OsteoCalVitFort usage.

**Conclusion:**

The results of the research indicate despite the high quality of OsteoCalVitFort supplement, there are no side effects which have been seen in other supplements.

## Background

There is little debate about calcium needs during lifespan for optimizing health in health of bone and out of bone [1, 2]. Many people are avoid or limit dairy products because of lactase intolerant, then they have no choice but to eat supplement [3–5]. Calcium supplements cause few, if any, side effects, unfortunately [6]. Side effects can sometimes occur, including gas, constipation, bloating, nausea/vomiting, loss of appetite, mental/mood changes, and bone/muscle pain. Vitamin D3 is used to facilitate absorption of the calcium in the gut [7, 8], and to facilitate calcium incorporation in the bones. Low vitamin D levels are associated with impairment of the active absorption of calcium [9]. Fortified Vitamin D3 with calcium without gastrointestinal side effect is a greatest effective factor to prevent bone loss [10–12]. OsteoCalVitFort seems more potent form of other Vitamin D and Calcium supplement (500 mg calcium and 400 I.U. of vitamin D3). The aim of this study was to evaluate side effects and satisfaction level of OsteoCalVitFort among people who consumed calcium supplement.

## Method

This study was single arm, prospective study that was done on 186 people who consumed Calcium supplement. Inclusion criteria were participants with age between 45 to 70 years old, in good health. Exclusion criteria were presence of any chronic illness such as chronic hepatic diseases, chronic kidney diseases, cardiac, hematologic, gastrointestinal, psychological problems, any diagnosed malignancy, history of active inflammatory illness, breast feeding, pregnancy, and history of kidney stone, hyperparathyroidism. After complete explanation of the study aim, informed consent from each participant was gathered.

Each participant received 1 pack that contains 60 OsteoCalVitFort tablet and used two tablet OsteoCalVitFort daily (1 tablet after breakfast and 1 after dinner). Side effects and satisfaction about OsteoCalVitFort consumption were assessed through case report form (CRF) by phone call.

### Ethical considerations

This study was proposed and approved by the Ethics-in-Research Commission of Endocrinology and Metabolism Research Institute, Tehran University of Medical Sciences. Also, it has been registered on the Iranian clinical trial registration (www.irct.ir) as IRCT201312031414N30.

### Statistical analysis

Data were analyzed by using the SPSS version 16 for Windows (SPSS Inc., Chicago, IL). The one–way ANOVA was used to determine the statistical significance of differences between the values for this study. Values of *p* < 0.05 were considered statistically significant.

## Result

The samples were composed of 166 (89.2 %) women and 20 (10.8 %) men with mean age of 52.78 ± 13.21, and 49.0 ± 16.50, respectively. The rate of low illiterate was 32.8 %, of illiterate was 1.07 %, of diploma was 36.2 %, and of high was 20.3 %.

The rate of constipating (8.0 %) and bloating (12.5 %) were decreased significantly after OsteoCalVitFort supplement intake (1.2 %, and 0.6 %, respectively). The percent of metallic taste in mouth at the first study (8.0 %) was significantly reduced compared to end of study (0.6 %). Significantly, the rate of tiredness (9.7 %), weakness (5.1 %), and loss of appetite (1.7 %) were decreased compared to OsteoCalVitFort supplement intake (0.6 %, 1.2 %, and 0.6 %, respectively). Significant decrease in bone/muscle pain changes was observed at the end of study (Table 1).

**Table 1:** Comparison of the previous and next effects of taking OsteoCalVitFort

Side effects	Before Practice	After Practice	*P*-value
Nausea/Vomiting	2.3*	4.9	0.22
Constipating	8.0	1.2	0.003
Gas	6.8	2.5	0.146
Diarrhea	0.6	1.2	1.000
Bloating	12.5	0.6	0.000
Loss of appetite	1.7	0.6	0.04
Dry Mouth	8.5	3.1	0.07
Metallic taste in mouth	8.0	0.6	0.001
Increased thirst/urine	6.8	1.2	0.02
Tiredness	9.7	0.6	0.001
Weakness	5.1	1.2	0.03
Headache	7.4	3.1	0.057
Bone/muscle pain	12.5	1.2	0.000

*%, *P*-value <0.05

Totally, 94 % of participants were satisfaction with using OsteoCalVitFort supplement.

## Discussion

Osteoporosis is major medical issues at the community level and can be lead to excessive [13–17]. A study in Iran has shown that Osteoporosis is an important health challenge and increases with age and changes in life style [18]. Dietary calcium is critical for the growth and development of the body skeleton and it seems plays a vital role in the prevention of osteoporosis [19]. Many people are avoided dairy products because of lactase intolerant. In these situations, calcium supplements can help them meet their calcium requirements [5].

OsteoCalVitFort contain high amounts of vitamin D with calcium. The role of vitamin D is bone preservation by improving the absorption of calcium from food and reduce fractures in older [18]. Most people who received Calcium, Vitamin D supplementation meet the gastrointestinal side effects and they are not satisfied for this obligatory administered by their physician. After consume of OsteoCalVitFort significant positive impact in constipating, bloating, metallic taste in mouth, thirst, tiredness, weakness, loss of appetite, and bone/muscle pain were observed.

The present study showed satisfaction level of OsteoCalVitFort intakes about 94 % of patients was satisfied. Consumers have multiple brands from which to choose but few assurances that the products are of high quality. Intake of OsteoCalVitFort can lead to Compensation amounts of Calcium and vitamin D. This supplement has no side effects. Satisfaction is a behavioral perspective on product consumer and it is too important that quality and satisfaction to be in a line.

## Conclusion

The results of this survey have shown despite the high quality of OsteoCalVitFort supplement, there are not side effects which have been seen in other supplements.
