# Erratum to: Calcium vitamin D3 supplementation in clinical practice: side effect and satisfaction

**DOI:** 10.1186/s40200-017-0321-7

**Published:** 2017-09-18

**Authors:** Maryam Sanaei, Mohammad Banasiri, Gita Shafiee, Mahsa Rostami, Saba Alizad, Mehdi Ebrahimi, Bagher Larijani, Ramin Heshmat

**Affiliations:** 1 Chronic Diseases Research Center, Endocrinology and Metabolism Population Sciences Institute, Tehran University of Medical Sciences, # 111, 19th St, North Kargar Ave, Tehran, Iran; 2 Orthopaedic surgery Department, Medical school, Golestan University of Medical Sciences, Tehran, Iran; 3 Endocrinology and Metabolism Research Center, Endocrinology and Metabolism Research Institute, Tehran University of Medical Sciences, Tehran, Iran

## Erratum

This article [1] was unintentionally published twice in this journal, by the same authors. The following should be considered the version of record and used for citation purposes: "Sanaei et al., Calcium vitamin D3 supplementation in clinical practice: side effect and satisfaction, Journal of Diabetes & Metabolic Disorders (2016) 15:5, 10.1186/s40200-016-0227-9". The duplicate "Sanaei et al., Calcium vitamin D3 supplementation in clinical practice: side effect and satisfaction, Journal of Diabetes & Metabolic Disorders (2016) 15:9, 10.1186/s40200-016-0231-0" is to be ignored. Editor-in-Chief Prof Bagher Larijani apologizes to the readers of the journal for not detecting the duplication during the publication process.
